# Surgical Synergy in Primary Open-Angle Glaucoma: Assessing Safety and Efficacy of Hydrus, iStent, and Gonioscopy-Assisted Transluminal Trabeculotomy in Glaucoma Management

**DOI:** 10.3390/jcm13247758

**Published:** 2024-12-19

**Authors:** Mohammad Zeyad Mohammad Ayoub, Ahmed Al-Nahrawy

**Affiliations:** 1 Croydon University Hospital, 530 London Road, Thornton Heath, London CR7 7YE, UK; 2 Western Eye Hospital, 153-173 Marylebone Road, London NW1 5QH, UK

**Keywords:** minimally invasive glaucoma surgery, Hydrus, iStent, GATT, primary open-angle glaucoma

## Abstract

**Background/Objectives**: This paper will compare the outcomes—safety and efficacy—of three minimally invasive glaucoma surgeries (MIGSs),the Hydrus Microstent, iStent, and Gonioscopy-Assisted Transluminal Trabeculotomy (GATT), for intraocular pressure (IOP) reduction in patients with primary open-angle glaucoma (POAG). **Methods**: A literature search of Ovid Medline and Embase identified studies evaluating the Hydrus, iStent, and GATT. Data on IOP reduction, medication use, and complications were analyzed. **Results**: Studies show the Hydrus, iStent, and GATT reduce IOP and medication burden in POAG patients, with some complications. For the Hydrus, studies showed 37.09% (27.5 ± 4.4 to 17.3 ± 3.7 mmHg) and 25% (16.8 to 12.6 mmHg) IOP reduction. Meanwhile, medication burden decreased from 2.5 ± 0.7 to 1.0 and from 2.1 to 1.15. For the iStent, studies showed a 36.39% (21.1 to 13.4 mmHg) and 8.19% (17.1 to 15.7 mmHg) IOP drop. Medication burden decreased from 2.87 to 1.24 and from 1.7 to 0.26. For GATT, studies showed a 49.33% (27.70 ± 10.30 to 14.04 ± 3.75) and 39.09% (26.40 ± 6.37 to 16.08 ± 2.38) IOP drop. Medication burden reduced from 3.73 ± 0.98 to 1.82 ± 1.47 and from 3.12 ± 0.80 to 0.45 ± 0.96. **Conclusions**: The Hydrus, iStent, and GATT are effective alternatives to trabeculectomy for mild to moderate POAG. They reduce and control IOP and dependence on medications with manageable safety profiles. In all three options, there were some clinically significant complications based on the *p*-value. For the Hydrus, it was PAS. For the iStent, they were PAS, FB sensation, IOP spikes, and microhyphema. For GATT, it was IOP spikes. However, further long-term studies, especially randomized controlled trials, are needed to support these results.

## 1. Introduction

**Figure 1:**
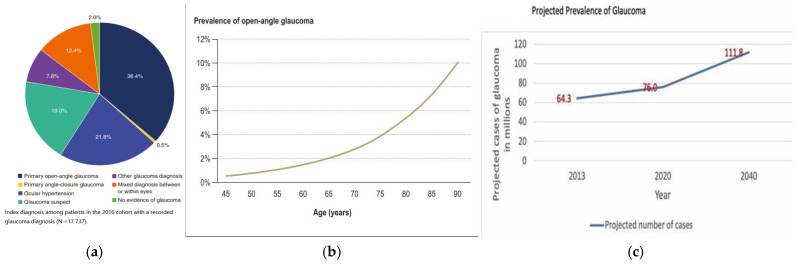
(**a**): Diagnosis among patients in the 2016 cohort with a recorded glaucoma diagnosis [5]; (**b**): prevalence of glaucoma between the ages of 45 and 80 [3]; and (**c**): projected number of glaucoma cases by 2040 in millions [1].

Glaucoma is a leading cause of blindness, mainly due to optic nerve damage and disk cupping, resulting in visual field loss. This damage is linked to the loss of retinal ganglion cells and raised intraocular pressure (IOP) [1]. Primary open-angle glaucoma (POAG) is more common than primary angle-closure glaucoma (PACG) [2]. This is seen in Figure 1a. This In 2010, glaucoma affected 2.1 million people globally, with 2.93% of Europeans aged 40 to 80 diagnosed [3]. The global prevalence is expected to rise from 76 million in 2020 to 111.8 million by 2040 [1] as highlighted in Figure 1c.

**Figure 2:**
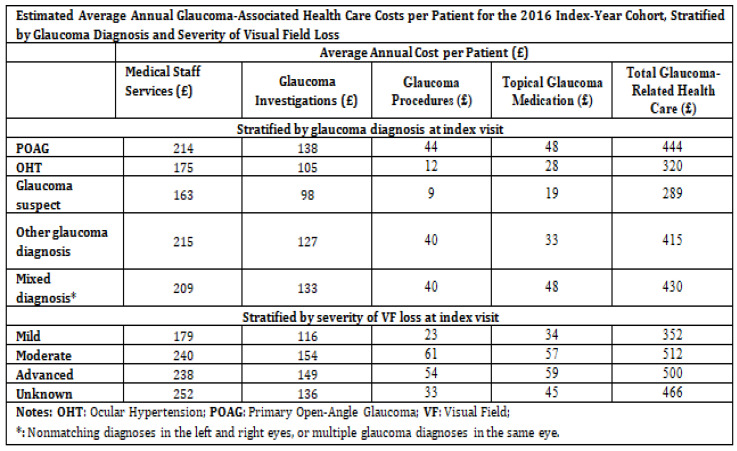
Estimated average annual glaucoma-associated health care costs per patient [5].

**Figure 3:**
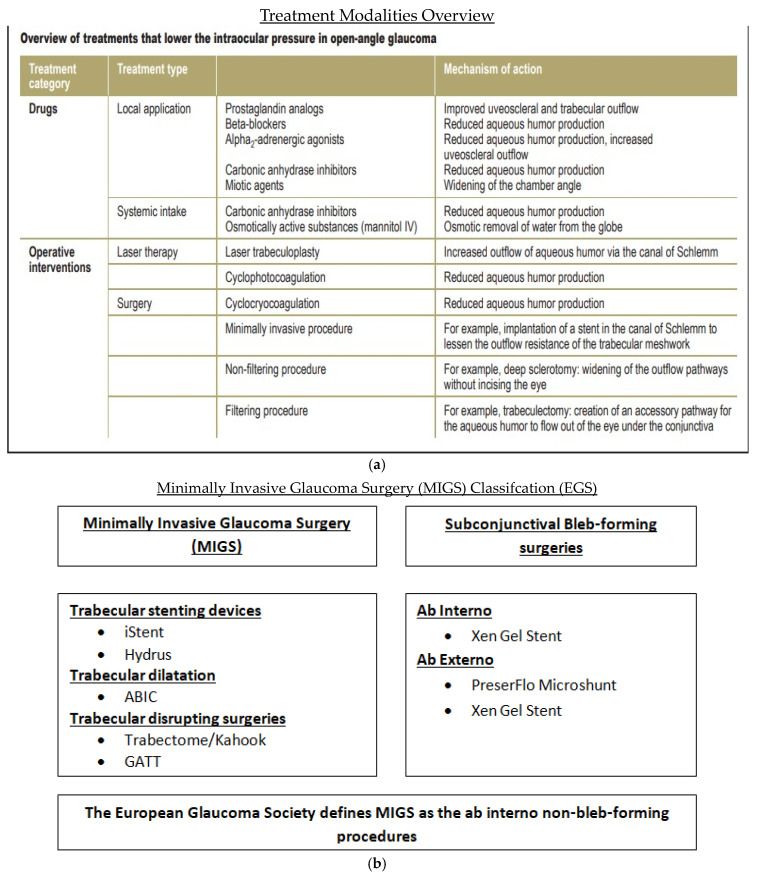
(**a**): Summary of the available treatments for POAG [3]; (**b**): classification of MIGS as per experts in the EGS.

In the last 40 years, glaucoma management has changed. Laser trabeculoplasty is considered as a safe and effective treatment for glaucoma, even before eye drops for some patients. The Tube Versus Trabeculectomy (TVT) study has also encouraged more use of glaucoma drainage implants (GDIs), even as a primary surgery. Microinvasive glaucoma surgery (MIGS) has increased treatment options further by lowering eye pressure through Schlemm’s canal, or by creating pathways to the suprachoroidal or subconjunctival spaces. Between 2008 and 2016, there was a 14.7% increase in the overall number of therapeutic glaucoma surgeries, from 294,990 to 338,230. It was seen that traditional glaucoma surgeries dropped by 11.7%, from 37,225 to 32,885 (*p* = 0.02). MIGS procedures increased by 426%, from 13,705 in 2012 to 58,345 in 2016 (*p* = 0.001). The number of trabeculectomies for Medicare patients dropped from 25,610 in 2008 to 18,925 in 2016 (*p* = 0.0001). Meanwhile, GDIs increased by 20.2% in Medicare patients, from 11,615 in 2008 to 13,960 in 2016 (*p* = 0.003) [4]. The economic burden of glaucoma is further highlighted in Figure 2. The various treatment options for glaucoma is shown in Figure 3a.

The primary goal of this study is to assess the efficacy and safety of the HMS, iStent, and GATT. These devices have been classified as MIGS as per experts in European Glaucoma Society and this is shown in Figure 3b.

## 2. Materials and Methods

The literature search for this study was conducted using the Ovid Embase and Ovid Medline databases. Search terms included glaucoma, open-angle glaucoma (OAG), intraocular pressure (IOP), minimally invasive glaucoma surgery (MIGS), iStent, Hydrus Microstent, and GATT. The search focused on comparison studies evaluating the efficacy and safety of these techniques.

Only studies published after 2015 were included to ensure the use of the most recent research. Selected studies mainly addressed the effectiveness and safety of the listed procedures.

**Figure 4:**
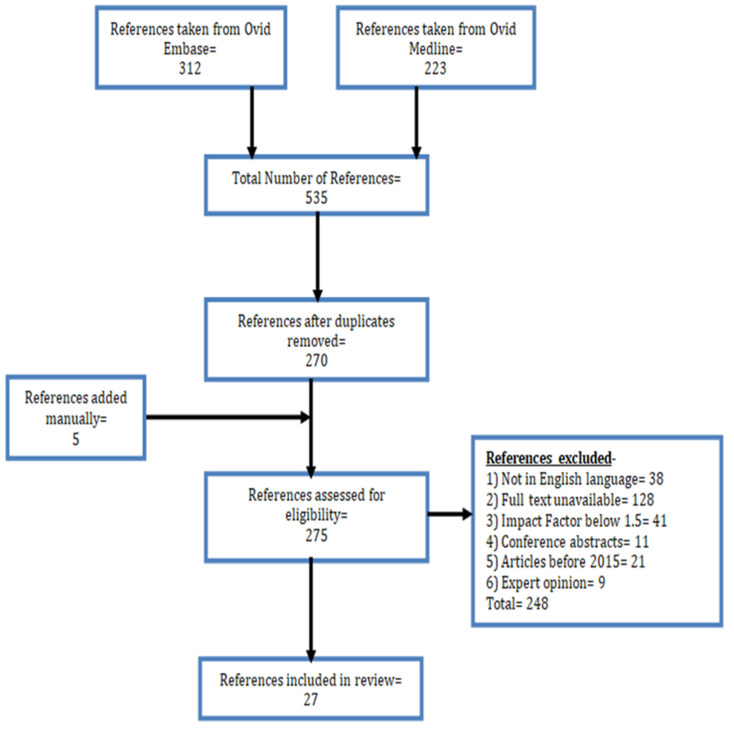
Flow diagram depicting the process for the selection of studies.

Study selection process is shown in Figure 4.

## 3. Results

### 3.1. Hydrus

**Figure 5:**
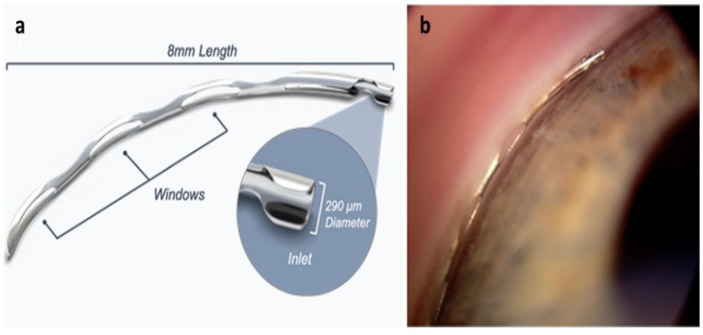
HMS (**a**) schematic and (**b**) gonioscopici mage [6].

The Hydrus Microstent (HMS) is a crescent-shaped device made from nitinol, measuring about 8 mm [6]. Its shape matches Schlemm’s canal (SC) and is placed in the eye using a preloaded injector, as per Figure 5a,b. After placement, the HMS expands within the SC, passing through the trabecular meshwork (TM) to improve the flow of aqueous humor from the anterior chamber (AC) into the SC, helping to lower eye pressure [7]. 

#### 3.1.1. The HORIZON Study [8]

This prospective, multicenter, single-masked randomized controlled trial compared the Hydrus Microstent (HMS) with cataract surgery (CS) versus CS alone in a 24-month trial involving 369 HMS + CS eyes and 187 CS eyes. At 24 months, IOP dropped from 25.5 ± 3.0 mmHg to 17.4 ± 3.7 mmHg in the HMS + CS group and from 25.4 ± 2.9 mmHg to 19.2 ± 3.8 mmHg in the CS group, with greater IOP reduction in the HMS + CS group (−7.6 ± 4.1 mmHg vs. −5.3 ± 3.9 mmHg). Medication use dropped more in the HMS + CS group (1.7 ± 0.9 to 0.3 ± 0.8) than in the CS group (1.7 ± 0.9 to 0.7 ± 0.9). Adverse effects included- uveitis (5.6% vs. 3.7%), conjunctivitis (5.7% vs. 7.0%), non-obstructive PAS (14.9% vs. 2.1%), and cystoid macular edema (2.2% vs. 2.1%).

#### 3.1.2. Five-Year Outcomes of the HORIZON Study [9]

This multicenter, randomized clinical trial compared five-year outcomes of the Hydrus Microstent (HMS) with cataract surgery (CS) versus CS alone. At five years, 308 HMS + CS eyes and 134 CS eyes were examined. In the HMS + CS group, diurnal IOP (DIOP) dropped from 25.5 ± 3.0 mmHg to 16.8 ± 3.1 mmHg, and medication use decreased from 1.7 ± 0.9 to 0.5 ± 0.9. In the CS group, DIOP decreased from 25.4 ± 2.9 mmHg to 17.2 ± 3.2 mmHg, and medication use dropped from 1.7 ± 0.9 to 0.9 ± 0.9. Adverse effects included visual field worsening (8.4% vs. 9.6%), prolonged steroid use (5.9% vs. 3.7%), and PAS (14.6% vs. 3.7%).

#### 3.1.3. Hydrus-II Study [10]

The HYDRUS II study was a prospective, single-masked, randomized controlled trial comparing outcomes of the Hydrus Microstent (HMS) with cataract surgery (CS) versus CS alone. In the HMS + CS group, pre-operative washed-out IOP was 26.3 ± 4.4 mmHg with 2.0 ± 1.0 medications. At 24 months, IOP dropped to 16.9 ± 3.3 mmHg with 0.5 ± 1.0 medications. In the CS group, pre-operative IOP was 26.6 ± 4.2 mmHg with 2.0 ± 1.1 medications, reducing to 19.2 ± 4.7 mmHg and 1.0 ± 1.0 medications at 24 months. Adverse effects were rare, with macular edema (1 vs. 2), PAS (6 vs. 1) and optic disk hemorrhage (1 vs. 0) among others.

#### 3.1.4. HMS vs. SLT in POAG-1 Year Results [11]

This prospective interventional case series compared the efficacy of Selective Laser Trabeculoplasty (SLT) with the Hydrus Microstent (HMS) in reducing IOP and medication use in patients with uncontrolled POAG. The SLT group (25 patients) saw an IOP decrease from 23.18 ± 2.15 mmHg to 15.9 ± 2.49 mmHg at 12 months, with medications dropping from 2.48 ± 0.92 to 2.0 ± 0.91. In the HMS group (31 patients), IOP reduced from 23.09 ± 5.08 mmHg to 16.5 ± 2.6 mmHg, and medications decreased from 2.29 ± 0.83 to 0.9 ± 1.04. Adverse effects in the HMS group included IOP spikes (6.45%) and transient visual acuity loss (9.68%).

#### 3.1.5. Hydrus vs. iStent: The COMPARE Study [12]

This prospective, multicenter, randomized clinical trial evaluated the efficacy of the Hydrus and iStent in 152 patients with OAG. In total, 75 patients were in the Hydrus group and 77 in the iStent group. Four patients (two from each group) missed follow-up appointments and were excluded. In the Hydrus group, pre-operative IOP was 19.0 ± 2.5 mmHg with 2.5 ± 0.7 medications, and the pre-operative WO-IOP was 27.5 ± 4.4 mmHg. At 12 months, IOP decreased to 17.3 ± 3.7 mmHg, and medications dropped to 1.0. In the iStent group, pre-operative IOP was 19.1 ± 3.6 mmHg with 2.7 ± 0.8 medications, and WO-IOP was 27.3 ± 4.2 mmHg. At 12 months, IOP fell to 18.1 ± 3.7 mmHg, and medications dropped to 1.7. Hydrus patients required fewer medications at 12 months, with 46.6% medication-free compared to 24.0% in the iStent group. Adverse effects included BCVA loss > 2 lines (2.7% vs. 1.3%), IOP spikes > 10 mmHg (4.1% vs. 5.2%), new cataract formation (2.6% vs. 1.3%), device obstruction (5.4% vs. 13.2%), and obstruction by PAS (6.8% vs. 0%).

#### 3.1.6. Phaco and MIGS Compared to Phaco Alone in OAG [13]

This retrospective case series examined post-operative outcomes of 297 eyes from 190 patients with OAG who underwent phaco alone, phaco + iStent, or phaco + Hydrus. Patients included 85 males and 105 females, with 148 eyes unaffected by OAG. In the OAG groups, 47 underwent phaco alone, 50 had phaco + iStent, and 52 had phaco + Hydrus. Pre-operative IOP was 13.6 mmHg in the phaco group, 17.9 mmHg in the phaco + iStent group, and 16.8 mmHg in the phaco + Hydrus group. At 24 months, IOP dropped to 12.6 mmHg, 13.8 mmHg, and 12.6 mmHg, respectively. Phaco + iStent and phaco + Hydrus showed greater IOP reduction than phaco alone. Medication use dropped by 0.3 in the phaco group, 0.7 in the phaco + iStent group, and 1.2 in the phaco + Hydrus group. At 12 months, 28.3% of phaco + iStent patients and 28.6% of phaco + Hydrus patients were medication-free, compared to 4.4% in the phaco-only group. IOP spikes (>30 mmHg) occurred in 17.0% of the phaco group, 3.9% of the phaco + iStent group, and 1.9% of the phaco + Hydrus group. Long-term complications included PAS and TM fibrosis in both stent groups.

Summary of the results of all 6 studies for Hydrus are shown in Table 1.

**Figure 6:**
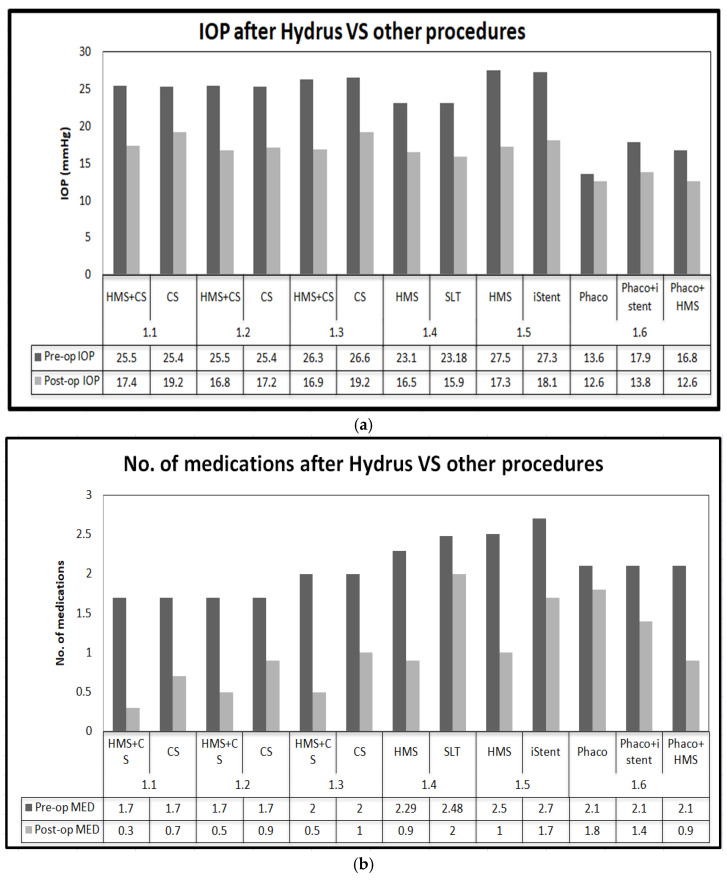
(**a**): Graph comparing IOP after Hydrus vs. other procedures; (**b**):graph comparing medication burden after Hydrus vs. other procedures.

Summary of outcomes of Hydrus are shown in Figure 6a,b.

### 3.2. ISTENT

**Figure 7:**
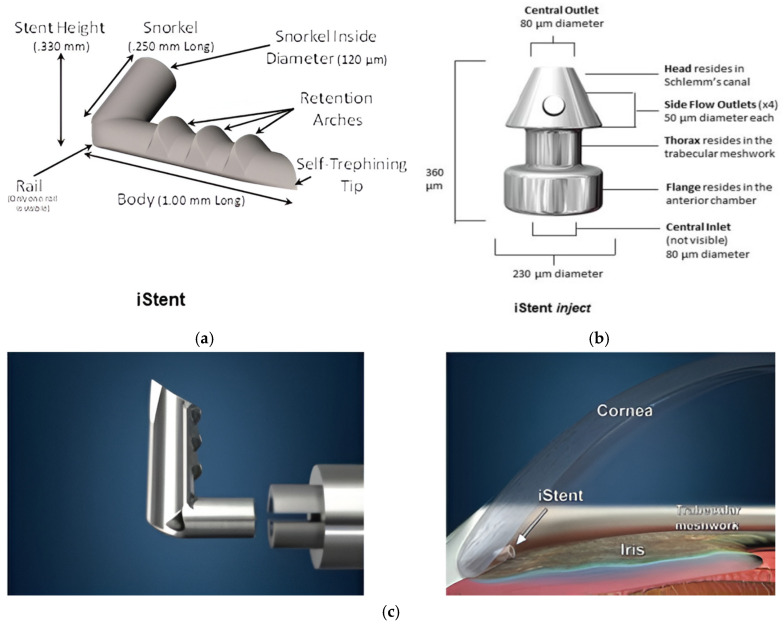
(**a**) iStent [14]; (**b**) iStent inject [14]; (**c**) iStent and placement location [15].

The first-generation iStent is made of titanium with a heparin coating. It is a 1.00 mm L-shaped stent inserted through an abinterno technique, using an injector guided by gonioscopy to pass through the trabecular meshwork (TM) and into Schlemm’s canal (SC). This approach allows for direct access to the anterior chamber [7,14].The second-generation iStent inject, inserted via a specially designed injector that holds two devices, is cone-shaped and improves fluid outflow through the TM into SC [7,14]. This is shown by Figure 7a–c.

#### 3.2.1. Standalone Implantation of iStent Inject ± iStent as Alternative to Trabeculectomy [16]

This retrospective study compared standalone stent implantation (iStent inject ± iStent) with trabeculectomy + Mitomycin C in 110 eyes (70 Multistent and 40 Trab) with mild-to-moderate OAG. Pre-op IOP was 21.1 mmHg (Multistent) and 22.3 mmHg (Trab) with 2.87 and 3.10 medications, respectively. Post-operatively, IOP dropped to 13.4–15.0 and 11.4–12.6 mmHg, while medications dropped to 1.24–1.62 and 0.15–0.95 in the Multistentand Trab group, respectively. No early complications were seen in Multistent, but about 30% in Trab group,includingIOP elevation, bleb failure, bleb leak, suture dehiscence, and shallow AC. Late complications were seen in both Multistent (6%) and Trab (33%). These included PAS and IOP elevation in the Multistent group. Meanwhile,in the Trab group, complications includedbleb failure, peripheral corneal thinning, blebitis, and clinically significant hypotony.

#### 3.2.2. Ab Interno-Implanted Trabecular Micro-Bypass in Primary Open-Angle Glaucoma—RCT [17]

This prospective, randomized, single-masked, multicenter trial involved 505 eyes to assess the safety and efficacy of the iStent inject with cataract surgery (CS) in mild-to-moderate POAG. In total, 387 eyes underwent iStent + CS and 118 had CS alone. Pre-op medicated IOP was 17.5 mmHg for both groups, with un-medicated IOP at 24.8 mmHg (iStent + CS) and 24.5 mmHg (CS-only). Post-operatively, IOP dropped to 17.1 mmHg (iStent + CS) and to 17.8 mmHg (CS-only). Medication use fell from 1.6 to 0.4 (iStent + CS) and 1.5 to 0.8 (CS-only). Adverse effects included BSCVA loss ≥ 2 lines (2.6% vs. 4.2%), PVD (2.6% vs. 4.2%), foreign body sensation (2.3% vs. 0%), and other complications like blurred vision, IOP increase, and corneal issues.

#### 3.2.3. Clinical Evaluation iStent with Phacoemulsification in Patients with OAG and Cataract [18]

This retrospective case series evaluated the safety and efficacy of the iStent with cataract surgery in 350 eyes with OAG and cataract. Pre-operative IOP was 19.13 mmHg with 1.19 medications. Post-operative IOP decreased to 14.95 mmHg at 6 months and 15.17 mmHg at 2 years. Medication use dropped to 0.61 drops on average. Complications included transient IOP spikes, additional tube shunt surgeries, and secondary glaucoma surgeries.

#### 3.2.4. Efficacy of iStent on IOP with Phaco vs. Phaco Alone in Glaucoma and Cataract Patients [19]

In a prospective, randomized, single-center trial of 80 patients with cataract and mild-moderate POAG, the iStent combined with cataract surgery (iStent + CS) was compared to cataract surgery alone (CS). The study found that in patients with an initial IOP less than 26 mmHg, the iStent + CS group achieved a greater reduction in IOP and medications, from 22.04 ± 1.64 to 15.57 ± 2.13 mmHg and from 1.32 ± 0.55 to 0.32 ± 0.55, respectively, compared to the CS-only group, from 20.93 ± 1.28 to 17.79 ± 2.50 mmHg and 1.03 ± 0.19 to 0.76 ± 0.69, respectively. For patients with initial IOP over 26 mmHg, the iStent + CS group also showed a greater IOP reduction—from 26.6 ± 1.09 to 17.06 ± 2.43 mmHg—compared to the CS-only group—from 26.00 ± 0.00 to 19.86 ± 2.19 mmHg—with medication use decreasing from 2.50 ± 0.89 to 0.88 ± 1.26 versus 1.86 ± 0.69 to 1.29 ± 0.76, respectively. Complications were minor and resolved within a week, including microhyphema and subconjunctival hemorrhage in the iStent + CS group, and corneal edema and inflammation in the CS-only group.

#### 3.2.5. Four-Year Outcomes of iStent Inject Stents in Patients with OAG on One Medication [20]

In a prospective multi-surgeon study of 57 patients with open-angle glaucoma, the efficacy of two iStent inject devices was evaluated over 48 months. Initially, patients had a mean medicated IOP of 19.5 mmHg on one medication and an unmedicated IOP of 24.4 mmHg. By 48 months, the unmedicated IOP decreased by 46% to 13.2 mmHg. At this time, 95% of patients had an IOP reduction of at least 20% without medication, 95% had IOP ≤ 18 mmHg, and 82% had IOP ≤ 15 mmHg without medication. Adverse effects included a loss of best-corrected visual acuity in two cases and IOP elevation in one case.

#### 3.2.6. Safety and Efficacy of the iStent Combined with Phacoemulsification [21]

The efficacy and safety of an iStent implantation cataract surgery in individuals with mild to moderate OAG and cataracts was investigated. The study design was a prospective, uncontrolled, interventional case series. In total, 54 patients were involved. The IOP values decreased from 17.1 ± 3.5 mmHg pre-operatively to 15.7 ± 2.2 mmHg post-operatively. The number of medications dropped from 1.7 ± 0.9 to 0.26. The adverse effects noted were subconjunctival hemorrhage, erythrocytes in the AC, corneal edema-associated increased IOP, and viral keratitis.

Summary of the results of all 6 studies for iStent are shown in Table 2.

**Figure 8:**
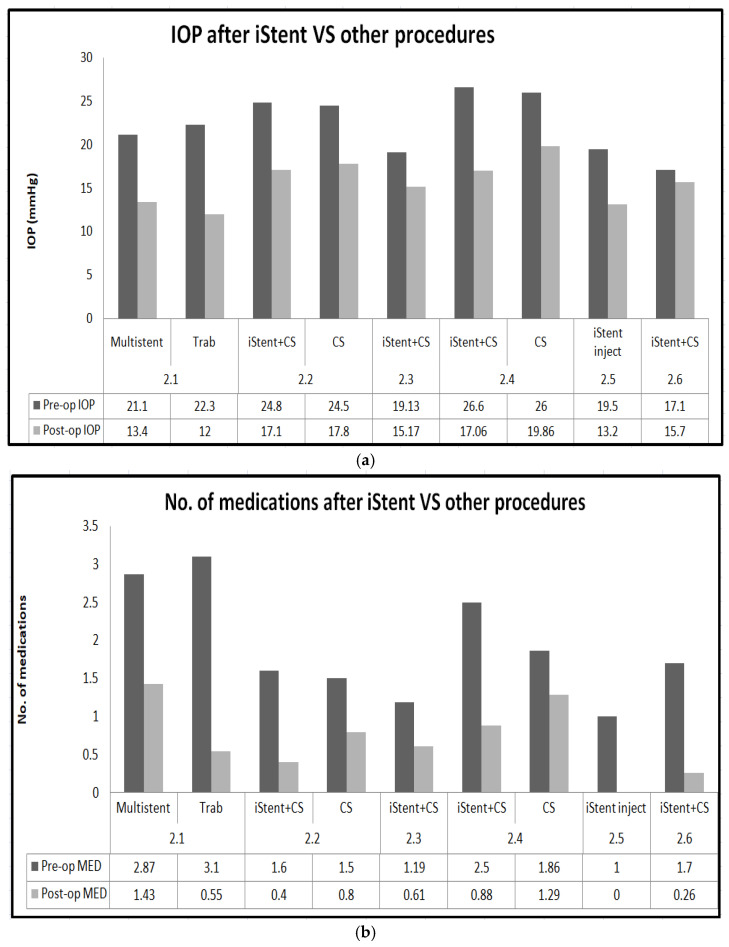
(**a**): Graph comparing IOP after iStent vs. other procedures; (**b**):graph comparing medication burden after iStent vs. other procedures.

Summary of outcomes of iStent are shown in Figure 8a,b.

### 3.3. GATT

**Figure 9:**
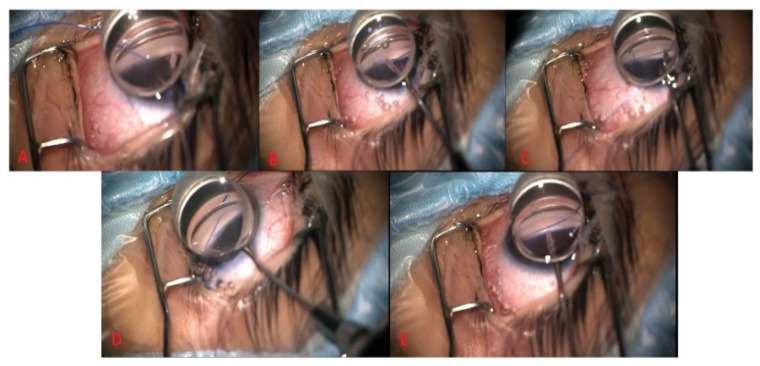
GATT steps: (**A**) Goniotomy incision via microsurgical blade. (**B**) Schlemm’s canal (SC) cannulation using 5/0 prolene suture. (**C**) Suture passed through SC. (**D**) Distal tip of suture is retrieved. (**E**) Distal end of suture externalized via microsurgical blade to exert traction on the suture, creating trabeculotomy [22].

GATT is a procedure that improves the flow of aqueous humor. It involves making an ab interno incision, guided by gonioscopy. Through this incision, a microcatheter or suture is passed to de-roof Schlemm’s canal. This reduces resistance to the outflow of aqueous humor [7]. The exact procedure is highlighted in Figure 9. 

#### 3.3.1. Gonioscopy-Assisted Transluminal Trabeculotomy in Younger to Middle-Aged Adults: One-Year Outcomes [23]

This retrospective case series assessed one-year outcomes of GATT in 56 eyes of 47 patients, with and without cataract surgery (CS). Pre-operative IOP was 27.70 ± 10.30 mmHg with 3.73 ± 0.98 medications. At 12 months, IOP dropped to 14.04 ± 3.75 mmHg. Medication use decreased from 3.73 ± 0.98 to 1.82 ± 1.47. Adverse events included hyphema, IOP spikes, corneal edema, BCVA loss, and lens-related changes.

#### 3.3.2. Four-Year Surgical Outcomes of Gonioscopy-Assisted Transluminal Trabeculotomy in Patients with Open-Angle Glaucoma [24]

This retrospective case series evaluated the effectiveness and safety of GATT in 74 eyes of 59 patients with open-angle glaucoma over four years. After excluding 28 eyes due to glaucoma reoperation, 31 eyes were analyzed. The average IOP decreased from 27.0 ± 10.0 mmHg to 14.8 ± 6.5 mmHg. Medication use also declined, from 3.2 ± 1.0 to 2.3 ± 1.0. Adverse effects included hyphema and IOP spikes.

#### 3.3.3. Gonioscopy-Assisted Transluminal Trabeculotomy (GATT) Combined Phacoemulsification Surgery: Outcomes at a 2-Year Follow-Up [25]

This study compared the effectiveness of GATT combined with phacoemulsification versus GATT alone for POAG with cataract. It involved 124 eyes: 58 with the combined procedure and 66 with GATT alone. In the combined procedure group, average IOP decreased from 26.40 ± 6.37 mmHg with 3.12 ± 0.80 to 14.61 ± 2.28 mmHg with 0.27 ± 0.71 medications at 12 months, and to 16.08 ± 2.38 mmHg with 0.45 ± 0.96 medications at 24 months. In the GATT-only group, IOP dropped from 27.54 ± 8.09 mmHg with 3.35 ± 0.64 medications to 15.57 ± 3.34 mmHg with 0.57 ± 1.22 medications at 12 months, and to 15.50 ± 3.40 mmHg with 0.95 ± 1.50 medications at 24 months. The combined procedure generally required fewer medications. Adverse effects included microhyphema, macrohyphema, IOP spikes, and supraciliary effusion.

#### 3.3.4. Comparison of Gonioscopy-Assisted Transluminal Trabeculotomy Versus Trabeculectomy with Mitomycin C in Patients with Open-Angle Glaucoma [26]

This retrospective, single-center study compared the effectiveness of GATT and MMC-augmented Trabeculectomy (TRAB) in lowering IOP for patients with uncontrolled open-angle glaucoma. The study included 110 eyes (61 GATT and 49 TRAB) and assessed outcomes at 18 months. Pre-operative IOP was 30.04 ± 7.5 mmHg for TRAB and 27.59 ± 4.70 mmHg for GATT, with medication counts of 3.08 ± 0.73 and 2.92 ± 0.91, respectively. At 18 months, TRAB achieved a mean IOP of 12.48 ± 4.58 mmHg, compared to 15.26 ± 3.47 mmHg GATT. Medication use decreased by 2.3 ± 1.4 for TRAB and 2.1 ± 1.5 for GATT. Complications included hypotony in TRAB cases and none in GATT, hyphaema in both GATT and TRAB cases, and IOP spikes in both procedures.

Summary of the results of all 4 studies for GATT are shown in Table 3.

**Figure 10:**
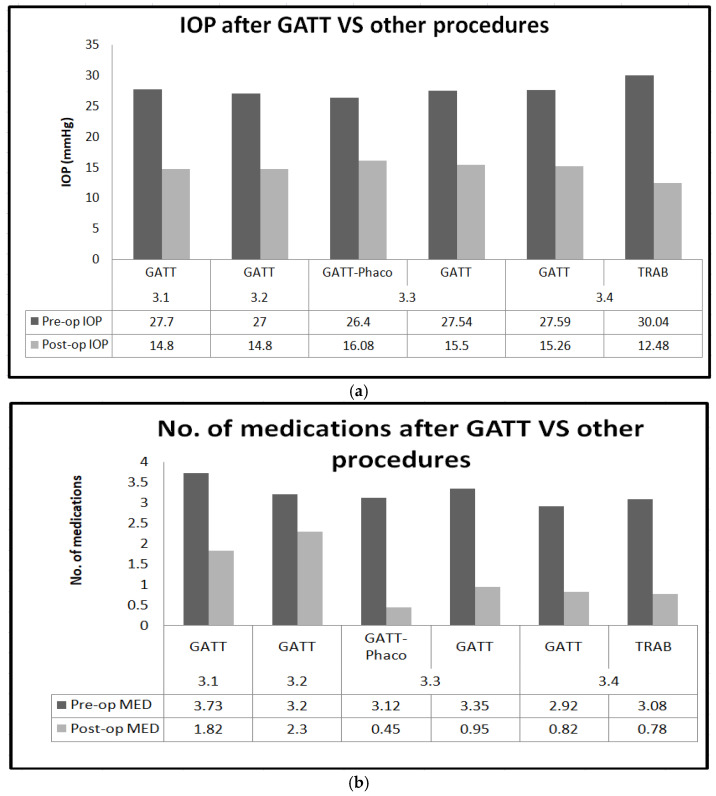
(**a**): Graph comparing IOP after GATT vs. other procedures; (**b**):graph comparing medication burden after GATT vs. other procedures.

Summary of outcomes of GATT are shown in Figure 10a,b.

Summary of all three (Hydrus, iStent and GATT) devices’ mechanism of action and implantation technique is mentioned in Table 4.

## 4. Discussion

### 4.1. IOP Reduction

Hydrus Microstent (HMS): The Hydrus Microstent (HMS) helps lower intraocular pressure (IOP) in patients with open-angle glaucoma (OAG), especially when combined with cataract surgery (CS). In the HORIZON study, patients who had both the HMS and CS saw their IOP drop from 25.5 mmHg to 17.4 mmHg over two years. In comparison, the CS-only group had a smaller drop, from 25.4 mmHg to 19.2 mmHg [8]. A five-year follow-up in the HORIZON trial showed that HMS + CS kept the IOP low at 16.8 mmHg, while CS-only was 17.2 mmHg [9].

iStent: The iStent also reduces IOP effectively when combined with cataract surgery. In the study by Samuelson, iStent + CS lowered IOP from 24.8 mmHg to 17.1 mmHg, while CS alone dropped IOP from 24.5 mmHg to 17.8 mmHg [27]. Ferguson found a similar IOP reduction from 19.13 mmHg to 15.17 mmHg after two years [18].

GATT (Gonioscopy-Assisted Transluminal Trabeculotomy): GATT is another good option for lowering IOP in patients with OAG. Wan et al. showed that when GATT was combined with CS, the IOP dropped from 26.40 mmHg to 16.08 mmHg over two years [25].

Device Comparison: Comparing the three devices, the HMS generally leads to a bigger drop in IOP, especially when used with cataract surgery. Ahmed et al. found that the HMS reduced IOP more than the iStent at 12 months. GATT also reduces IOP well, particularly for patients with higher baseline IOP. However, the HMS seems to offer the most long-term IOP control.

The variability in baseline intraocular pressure (IOP) across the included studies poses a significant challenge when comparing the efficacy of the three MIGS devices. It is well established that patients with higher baseline IOP levels tend to experience more substantial absolute reductions in IOP following surgical interventions. This can create a misleading perception of the effectiveness of certain devices if these comparisons are made without appropriate adjustments. For instance, studies involving the Hydrus Microstent often included participants with elevated baseline IOP, potentially exaggerating the observed IOP reductions compared to studies on the iStent or GATT, which involved lower baseline IOP levels. This variability complicates meta-analyses that aggregate heterogeneous data, highlighting the importance of future studies employing standardized methodologies, including statistical adjustments for baseline IOP, to enable more accurate cross-device comparisons. Where possible, we have noted whether the included studies made such adjustments, and emphasize that differences in baseline IOP should be carefully considered when interpreting the relative outcomes among these devices.

A notable limitation of this analysis is the lack of long-term data for the iStent and GATT, particularly in comparison to the extensive follow-up available for the Hydrus Microstent through studies such as the HORIZON trial. This disparity limits the ability to draw robust conclusions regarding the relative long-term efficacy and safety of these devices. While short- to mid-term outcomes for the iStent and GATT are promising, comparable longitudinal studies are essential to better understand their sustained performance, durability, and complication profiles over extended periods. Future research should prioritize long-term randomized controlled trials to ensure a balanced and comprehensive assessment of these MIGS devices with accurate comparative results.

### 4.2. Reduction in Medications

A reduction in the number of glaucoma medications post-surgery is a significant outcome reported across the studies analyzed. However, differences in patient compliance with prescribed medications could influence the observed results, as non-adherence can lead to variable intraocular pressure (IOP) outcomes. While some studies attempted to standardize medication regimens before and after surgery, such as employing washout periods or specific protocols, this was not consistently reported. Moreover, there are currently no widely accepted statistical tests available to specifically quantify or adjust for the effects of non-compliance in this context, which adds another layer of complexity to interpreting these results.

Hydrus Microstent (HMS): The HMS also helps reduce the need for glaucoma medications. In the HORIZON study, patients using the HMS with CS had their medication use drop from 1.7 to 0.3 medications over two years [8].At five years, Ahmed et al. reported that the medication use dropped to 0.5, compared to 0.9 in the CS-only group [9].

iStent: The iStent also reduces medication use. In Samuelson et al., the iStent with CS lowered medication use from 1.6 to 0.4, while CS alone reduced it to 0.8 [27]. Ferguson et al. found a similar reduction, from 1.19 to 0.61 medications after two years. However, the iStent tends to have a smaller reduction in medication use compared to the HMS [18].

GATT: GATT shows good results in reducing medication use, although it varies across studies. Wan et al. reported that GATT with CS reduced medication use from 3.12 to 0.45 over two years [25]. GATT reduces medications, but it may not be as effective as the HMS in achieving medication-free status.

Device Comparison: The HMS shows the biggest and most lasting reduction in medication use. The COMPARE study found that 46.6% of HMS patients were medication-free at 12 months, compared to 24.0% in the iStent group. GATT is effective but tends to leave more patients needing medication compared to the HMS and iStent.

Future research should focus on implementing standardized medication protocols and adherence monitoring while exploring statistical methods to address the potential impact of non-compliance on surgical outcomes.

### 4.3. Complications

Each MIGS device carries specific risks of complications influenced by both device design and procedural factors. For the Hydrus Microstent, the incidence of peripheral anterior synechiae (PAS) may be attributed to its placement within Schlemm’s canal, where close proximity to trabecular meshwork tissue can induce adhesion. For the iStent, foreign body sensation could result from the device’s interaction with the conjunctiva or iris during implantation, though reports of this complication are generally rare and transient. In GATT, intraocular pressure (IOP) spikes are commonly associated with transient obstruction of the trabecular outflow pathway, often due to post-operative inflammation or debris. Comparatively, traditional surgical options like trabeculectomy and tube shunts are associated with higher rates of significant complications, such as hypotony, bleb-related infections, and vision-threatening issues like choroidal detachment. While MIGS devices offer a more favorable safety profile overall, the specific complications noted for each device require further investigation to identify patient-specific risk factors and optimize surgical techniques.

Hydrus Microstent (HMS): The HMS is generally safe but does have some complications. In the HORIZON study, complications included peripheral anterior synechiae (PAS) in 14.9% of patients, compared to 2.1% in the CS-only group. Other issues included uveitis and cystoid macular edema [8]. The five-year follow-up by Ahmed et al. found a higher rate of PAS in the HMS group [9].

The incidence of peripheral anterior synechiae (PAS) was notably higher in the Hydrus Microstent group compared to controls, as reported in studies such as the HORIZON trial. However, the degree of PAS was not uniformly graded across the studies included in this analysis, limiting our ability to quantify its direct impact on clinical outcomes such as intraocular pressure (IOP) control or visual acuity. While some studies noted PAS as a non-obstructive finding with minimal clinical relevance, others suggested that extensive PAS could interfere with aqueous outflow and potentially compromise the efficacy of IOP reduction. Future studies should incorporate standardized grading systems for PAS and evaluate its correlation with both short- and long-term clinical outcomes to better understand its significance.

iStent: The iStent has a relatively low complication rate. Samuelson et al. reported minor issues like transient IOP spikes and device obstruction [27]. Kozera et al. highlighted complications like microhyphema and corneal edema [19].

GATT: Wan et al. (2023) also noted hyphema, IOP spikes, and supraciliary effusion in some cases [25].

IOP spikes were a notable complication associated with GATT. Studies reported that IOP spikes occurred in approximately 17.2% of eyes undergoing GATT combined with phacoemulsification surgery and in 36% of eyes with standalone GATT, a statistically significant difference (*p*< 0.05). These spikes were typically managed with glaucoma medications or by releasing aqueous humor through paracentesis from the surgical site. In rare cases, refractory IOP spikes required more invasive interventions, such as secondary surgical procedures. For example, 3% of GATT cases underwent reoperation due to persistent IOP elevation or associated complications like massive hyphema. Although IOP spikes are transient in most instances, their management underscores the importance of close post-operative monitoring and tailored therapeutic strategies.

Device Comparison: When comparing complications, all three options had some clinically significant complications based on the *p*-value. For the Hydrus, it was PAS. For the iStent, they were PAS, FB sensation, IOP spikes, and microhyphaema. For GATT, it was IOP spikes.

## 5. Limitations of This Review

The inclusion of both prospective and retrospective studies introduces the potential for selection bias, particularly in retrospectively obtained case series where patient inclusion criteria and data collection protocols may vary. To address this, we carefully reviewed the methodologies of the included studies and ensured that each met predefined eligibility criteria to minimize heterogeneity. Where possible, we prioritized data from prospective randomized controlled trials (RCTs) for comparative conclusions, as these studies inherently reduce bias through controlled patient selection and standardized protocols. However, we acknowledge that retrospective data may introduce unavoidable biases due to factors such as incomplete records or non-standardized follow-up intervals. This limitation is discussed as a caveat in the interpretation of pooled results, emphasizing the need for cautious extrapolation and the importance of conducting more RCTs in the future.

The studies included in this analysis often employed exclusion criteria to ensure homogeneity within their patient populations. Common exclusions included advanced glaucoma, prior glaucoma surgeries, and significant ocular comorbidities such as uveitis, corneal opacities, or retinal diseases. While these criteria enhance the internal validity of individual studies, they may limit the generalizability of the findings to broader clinical populations, particularly patients with more severe disease or complex ocular histories. It is important to note that these exclusions may underestimate the variability in real-world outcomes where such comorbidities are more prevalent. Future research should aim to include a more diverse range of patients to reflect the complexities of clinical practice better and provide a clearer understanding of how these devices perform across different patient subgroups.

An important limitation of this analysis is the limited discussion of patient-specific characteristics that may influence the choice of MIGS devices. Factors such as baseline glaucoma severity, presence of ocular or systemic comorbidities, and individual patient preferences are critical in guiding clinical decision-making. For instance, patients with mild-to-moderate glaucoma may benefit more from less invasive devices like the iStent, while those with higher baseline intraocular pressure or more advanced disease might require interventions like GATT or the Hydrus. Additionally, conditions such as uveitis or angle abnormalities could impact device selection and outcomes. The included studies provided limited data on these aspects, underscoring the need for future research to incorporate patient-centric factors and stratified analyses to improve the applicability of findings to diverse clinical populations.

A further limitation of this analysis is the inconsistency in the application of multiple testing corrections, such as Bonferroni adjustment, across the included studies. This inconsistency limits our ability to uniformly account for Type I errors when synthesizing findings from multiple comparisons. To address this, we emphasize the importance of future research adopting consistent use of multiple testing corrections to ensure the reliability and robustness of reported outcomes. Incorporating such methods will enhance the accuracy of statistical interpretations and reduce the likelihood of false-positive findings in future studies.

## 6. Conclusions

In conclusion, studies show that MIGS devices like the Hydrus Microstent, the iStent, and GATT effectively reduce intraocular pressure (IOP) and the need for medications in patients with open-angle glaucoma (OAG). When combined with cataract surgery, both the Hydrus and iStent significantly improve IOP and decrease medication use, enhancing patients’ quality of life. GATT also offers promising results. In all three options, there were some clinically significant complications based on the *p*-value. For the Hydrus, it was PAS. For the iStent, they were PAS, FB sensation, IOP spikes, and microhyphaema. For GATT, it was IOP spikes. These clinically significant complications need to be investigated further. These studies highlight that all three are valuable options for managing mild to moderate glaucoma.

The choice of which device to offer a patient should be made on a case-by-case basis after thorough counseling. This process should involve discussing the predicted risk–benefit profile of each device to help the patient make an informed decision based on their preferences. Additionally, other factors, such as the surgeon’s level of experience with a specific device, its availability, and institutional supply chain considerations, may also play a role in influencing device selection.

While the current studies provide valuable insights into the efficacy and safety of MIGS devices, there remain critical gaps that future long-term randomized controlled trials (RCTs) should address. Beyond traditional outcomes like intraocular pressure (IOP) reduction and medication burden, future studies should prioritize patient-reported outcomes and quality of life (QoL) metrics to understand the real-world impact of these interventions better. Another point that remains crucial to investigate is cost-effectiveness analyses to evaluate the economic implications of these devices, particularly in terms of reducing health care costs through lower medication use and fewer follow-up procedures. Investigating device durability, long-term safety profiles, and outcomes in diverse patient populations, including those with advanced glaucoma or ocular comorbidities, will also provide more comprehensive guidance for clinical decision-making.

## Figures and Tables

**Table 1:** Studies and all the relevant findings—Hydrus.

3.1.1: The HORIZON study [8]								
Study type: Prospective, randomized controlled trial				Duration of study: 2 years				
No of Centers: 26 investigational sites in the US				Total Population: 369 eyes (HMS); 187 eyes (CS)				
	Pre-op	Post-op (2 yr)	Decrease	Adverse effects—(% of the popn)		HMS + CS	CS	*p*-Value
HMS + CS (369)	IOP (mmHG) = 25.5 ± 3.0 MED = 1.7 ± 0.9	IOP (mmHG) = 17.4 ± 3.7 MED = 0.3 ± 0.8	IOP = 31.8% MED = −1.4	Uveitis/iritis		5.6	3.7	0.24
				Conjunctivitis		5.7	7.0	0.38
				Layered hyphema		0.5	0.5	1.00
				Corneal abrasion		1.1	0	0.3
				Corneal edema		1.4	0	0.18
CS (187)	IOP (mmHG) = 25.4 ± 2.9 MED = 1.7 ± 0.9	IOP (mmHG) = 19.2 ± 3.8 MED = 0.7 ± 0.9	IOP = 24.4% MED = −1	Non-obstructive PAS		14.9	2.1	1.9 × 10^−9^
				Obstructive PAS		3.8	0	0.003
				Cystoid macular edema		2.2	2.1	0.37
				Subconjunctival hemorrhage		2.4	0	0.02
3.1.2. 5-year outcomes of The HORIZON study [9]								
Study type: Multicenter Randomized Clinical Trial				Duration of study:5 years				
No of Centers: 26 investigational sites in the US				Total Population: 308/369 (HMS); 134/187 (CS)				
	Pre-op	Post-op	Decrease	Adverse effects—(% of the popn)		HMS + CS	CS	*p*-Value
HMS + CS (308)	IOP (mmHG) = 25.5 ± 3.0 MED = 1.7 ± 0.9	IOP (mmHG) = 16.8 ± 3.1 MED = 0.50 ± 0.9	IOP = 34.1% MED = −1.2	Visual field MD ≥ 2.5 deterioration		8.4	9.6	0.68
				Inflammation needing steroid use		5.9	3.7	0.18
CS (134)	IOP (mmHG) = 25.4 ± 2.9 MED = 1.7 ± 0.9	IOP (mmHG) = 17.2 ± 3.2 MED = 0.9 ± 0.9	IOP = 32.23% MED = −0.8	Peripheral anterior synechia		14.7	3.7	4.7× 10^−7^
				BCVA loss ≥ 2 ETDRS lines		1.9	2.1	0.69
3.1.3. Hydrus-II Study [10]								
Study type: prospective, randomized controlled trial				Duration of study:2 years				
No of Centers: 7 European investigational sites				Total Population: 100				
	Pre-op	Post-op	Decrease	Adverse effects—(no. of the popn)		HMS + CS	CS	*p*-Value
HMS + CS (50)	IOP (mmHG) = 26.3 ± 4.4 MED = 2.0 ± 1.0	IOP (mmHG) = 16.9 ± 3.3 MED = 0.5 ± 1.0	IOP = 35.7% MED = −1.5	Retinal detachment		0	1	1.0
				Wound dehiscence		0	1	1.0
				Ant. ischemic optic neuropathy		0	1	1.0
CS- (50)	IOP (mmHG) =26.6 ± 4.2 MED = 2.0 ± 1.1	IOP (mmHG) = 19.2 ± 4.7 MED = 1.0 ± 1.0	IOP = 27.8% MED = −1.0	Loss of BCVA > two lines		0	3	0.24
				Macular edema		1	2	1.0
				Focal PAS		6	1	0.11
				Optic disk hemorrhage		1	0	1.0
3.1.4. HMS vs. SLT in POAG-1 year results [11]								
Study type: Prospective interventional case-series				Duration of study: 1 year				
No of Centers: 2 sites				Total Population: 56–31 (HMS), 25 (SLT)				
	Pre-op	Post-op	Decrease	Adverse effects (% of the popn)		HMS	SLT	*p*-Value
HMS (31)	IOP (mmHG) = 23.09 ± 5.08 MED = 2.29 ± 0.83	IOP (mmHG) = 16.5 ± 2.6 MED = 0.9 ± 1.04	IOP = 28.5% MED = −1.39	IOP spikes		6.45	0	0.5
SLT (25)	IOP (mmHG) = 23.18 ± 2.15 MED = 2.48 ± 0.92	IOP (mmHG) = 15.9 ± 2.49 MED = 2.0 ± 0.91	IOP = 31.4% MED = −0.48	Transient VS loss > 2 lines		9.68	0	0.25
3.1.5. Hydrus vs. iStent: The COMPARE Study [12]								
Study type: Prospective, multicenter, randomized clinical trial				Duration of study:1 year				
No of Centers: 12 investigational sites in 9 countries				Total Population: 152 eyes—75 HMS, 77 iStent				
	Pre-op	Post-op	Decrease	Adverse effects (% of the popn)		HMS	iStent	*p*-Value
HMS (75)	IOP (mmHG) = 27.5 ± 4.4 MED = 2.5 ± 0.7	IOP (mmHG) = 17.3 ± 3.7 MED = 1.0	IOP = 37.1% MED = −1.5	BCVA loss > 2 lines		2.7	1.3	1.0
				IOP spike > 10 mmHg		4.1	5.2	1.0
iStent (77)	IOP (mmHG) = 27.3 ± 4.2 MED = 2.7 ± 0.8	IOP (mmHG) = 18.1 ± 3.7 MED = 1.7	IOP = 33.7% MED = −1.0	New cataract		2.6	1.3	1.0
				Device obstruction for iris		5.4	13.2	0.15
				Obstruction for PAS		6.8	0	0. 027
3.1.6. Phaco and MIGS compared to Phaco alone in OAG [13]								
Study type: Retrospective consecutive case series				Duration of study: 2 years				
No of Centers: 1 private surgical center in Australia				Total Population: 149 = 47 phaco, 50 phaco–iStent, 52 phaco–Hydrus				
	Pre-op	Post-op	Decrease	Adverse effects—(% of the popn)	Phaco	P + iStent	P + HMS	*p*-Value
Phaco (47)	IOP (mmHG) = 13.6 MED = 2.1–2.6	IOP (mmHG) = 12.6 MED = 1.8	IOP = 7.4% MED = −0.3 ± 0.1	IOP spike	17.0	1.9	3.9	0.0088
P + iStent (50)	IOP (mmHG) = 17.9 MED = 2.1 to 2.6	IOP (mmHG) = 13.8 MED = 1.4	IOP = 22.9% MED = −0.7 ± 0.2	PAS	N/A	ND	ND	N/A
P + HMS (52)	IOP (mmHG) = 16.8 MED = 2.1 to 2.6	IOP (mmHG) = 12.6 MED = 0.9	IOP = 25% MED = −1.2 ± 0.1	Trabecular meshwork fibrosis	N/A	ND	ND	N/A

Abbreviations: HMS—Hydrus Microstent; CS—cataract surgery; Phaco/P—phacoemulsification; SLT—Selective Laser Trabeculoplasty; IOP—intraocular pressure; MED—medication; Popn—population.

**Table 2:** Studies and all the relevant findings—iStent.

3.2.1. Standalone Implantation of iStent inject ± iStent as alternative to Trabeculectomy [16]							
Study type: Retrospective, consecutive study				Duration of study: 2 years			
No of Centers: 1 center (1 surgeon)				Total Population: 110 eyes—70 Multistent, 40 Trab			
	Pre-op	Post-op	% Decrease	Adverse effects—(no of the popn)	Multistent	Trab	*p*-value
Multistent (70)	IOP (mmHG) = 21.1 MED = 2.87	IOP (mmHG) = 13.4 to 15.00 MED = 1.24 to 1.62	IOP = 36.5% MED = −1.63 to −1.25	Early			
				IOP elevation	0	5	0.057
				Bleb failure	0	3	0.15
				Bleb leak	0	2	0.35
				Suture dehiscence	0	1	0.63
				Shallow AC	0	1	0.63
Trab (40)	IOP (mmHG) = 22.3 MED = 3.10	IOP (mmHG) = 11.4 to 12.6 MED = 0.15 to 0.95	IOP = 48.9% MED = −2.95 to −2.15	Late			
				PAS	3	0	0.036
				IOP elevation	1	0	0.99
				Bleb failure	0	9	0.0001
				Corneal thinning	0	2	0.35
				Blebitis	0	1	0.63
				Hypotony	0	1	0.63
3.2.2. Ab Interno-Implanted Trabecular Micro-Bypass in Primary Open-Angle Glaucoma—RCT [17]							
Study type: Prospective, multicenter, randomized clinical trial				Duration of study: 2 years			
No of Centers: 1/41 sites				Total Population: 505 eyes—387 iStent, 118 CS			
	Pre-op	Post-op	% Decrease	Adverse effects—(% of the popn)	iStent + CS	CS	*p*-value
iStent + CS (387)	IOP (mmHG) = 24.8 ± 3.3 MED = 1.6 ± 0.8	IOP (mmHG) = 17.1 ± 3.6 MED = 0.4 ± 0.8	IOP = 31.1% MED = −1.2	Loss of BSCVA ≥ 2 lines	2.6	4.2	0.21
				PVD	2.6	4.2	0.21
				FB sensation	2.3	0	0.0015
				Blurred vision	2.3	1.7	0.078
				EO inflammation	2.3	1.7	0.078
CS (118)	IOP (mmHG) = 24.5 ± 3.1 MED = 1.5 ± 0.7	IOP (mmHG) = 17.8 ± 3.5 MED = 0.8 ± 1.0	IOP = 27.4% MED = −0.7	Epi-retinal membrane	2.3	2.5	0.17
				IOP increase ≥10 mmHg	2.1	0.8	0.032
				Vitreous floaters	2.1	2.5	0.42
				Corneal abrasion	2.1	3.4	0.32
				Corneal opacity	1.0	2.5	0.63
3.2.3. Clinical evaluation iStent with phacoemulsification in patients with OAG and cataract [18]							
Study type: Retrospective, consecutive case series				Duration of study: 2 years			
No of Centers: 1				Total Population: 350 eyes			
	Pre-op	Post-op	% Decrease	Adverse effects—(no. of the popn)		iStent + CS	
iStent + CS (350)	IOP (mmHG) = 19.13 ± 6.34 MED = 1.19 ± 1.00	IOP (mmHG) = 15.17 ± 3.53 MED = 0.61 ± 0.96	IOP = 20.7% MED = −0.58	Transient IOP spikes of ≥15 mmHg		31	
				Additional tube shunt surgeries		2	
				Secondary glaucoma surgeries		3	
3.2.4.Efficacy of iStent on IOP with Phaco vs. Phaco alone in glaucoma and cataract patients [19]							
Study type: Prospective, single center, randomized clinical trial				Duration of study: 2 years			
No of Centers: 1				Total Population: 80–44 iStent + CS, 36 cataract surgery			
	Pre-op	Post-op	% Decrease	Adverse effects (no. of the popn)	iStent + CS	CS	*p*-value
iStent + CS (44)	IOP (mmHG) = 26.6 ± 1.09 MED = 2.50 ± 0.89	IOP (mmHG) = 17.06 ± 2.43 MED = 0.88 ± 1.26	IOP = 35.9% MED = −1.62	Microhyphema	5	0	0.02
				Subconjunctival hemorrhage	1	0	0.29
CS (36)	IOP (mmHG) = 26.0 ± 0.0 0 MED = 1.86 ± 0.69	IOP (mmHG) = 19.86 ± 2.19 MED = 1.29 ± 0.76	IOP = 23.6% MED = −0.57	Corneal edema	0	1	0.49
				Corneal inflammation	0	1	0.49
3.2.5.Four-year outcomes of iStent inject stents in patients with OAG on one medication [20]							
Study type: Prospective, interventional, multi-surgeon study				Duration of study: 2 years			
No of Centers: 1 center				Total Population: 57 eyes			
	Pre-op	Post-op	% Decrease	Adverse effects (no. of the pon)		iStent	
iStent inject (57)	IOP (mmHG) = 19.5 ± 1.5 MED = 1	IOP (mmHG) = 13.2 ± 1.6 MED = 0	IOP = 32.3% MED = −1	BCVA loss > 1 line		2	
				IOP elevation		1	
3.2.6. Safety and Efficacy of the iStent combined with phacoemulsification [21]							
Study type: Prospective, interventional case series				Duration of study: 3 years			
No of Centers: 1				Total Population: 54 eyes of 52 people			
	Pre-op	Post-op	% Decrease	Adverse effects (no. of popn)		iStent + CS	
iStent + CS (54)	IOP (mmHG) = 17.1 ± 3.5 MED = 1.7 ± 0.9	IOP (mmHG) = 15.7 ± 2.2 MED = 0.26	IOP = 8.2% MED = −1.44	Subconjunctival hemorrhage		1	
				Erythrocytes in AC		5	
				Corneal edema		1	
				Viral Keratitis		1	

Abbreviations: Trab—trabeculectomy; CS—cataract surgery; IOP—intraocular pressure; MED—medication; Popn—population.

**Table 3:** Studies and all the relevant findings—GATT.

3.3.1. Gonioscopy-Assisted Transluminal Trabeculotomy in Younger to Middle-Aged Adults: One-Year Outcomes [23]							
Study type: Retrospective case series				Duration of study: 1 year			
No of Centers: 2				Total Population: 56 eyes from 47 patients			
	Pre-op	Post-op	% Decrease	Adverse effects—(No of the popn)			GATT
GATT (56)	IOP (mmHG) = 27.70 ± 10.30 MED = 3.73 ± 0.98	IOP (mmHG) = 14.04 ± 3.75 MED = 1.82 ± 1.47	IOP = 49.3% MED = −1.91	Hyphema			40
				IOP spike			26
				Corneal edema			9
				BCVA loss > 2 lines			9
				Lens-related changes			3
3.3.2. Four-year Surgical Outcomes of Gonioscopy-assisted Transluminal Trabeculotomy in Patients with Open-Angle Glaucoma [24]							
Study type: Retrospective case series				Duration of study: 4 years			
No of Centers: 1 Surgeon, 1 center				Total Population: 74 eyes			
	Pre-op	Post-op	% Decrease	Adverse effects—(No of the popn)			GATT
GATT (74)	IOP (mmHG) = 27.0 ± 10.0 MED = 3.2 ± 1.0	IOP (mmHG) = 14.8 ± 6.5 MED = 2.3 ± 1.0	IOP = 45.2 MED = −0.9	Hyphema			27
				Significanthyphema			1
				IOP spike			16
3.3.3.Gonioscopy-assisted Transluminal Trabeculotomy (GATT) combined phacoemulsification surgery: Outcomes at a 2-year follow-up [25]							
Study type: Consecutive case series study				Duration of study: 2 years			
No of Centers: 1				Total Population: 124 eyes—Gatt + Phaco: 58, GATT: 66			
	Pre-op	Post-op	% Decrease	Adverse effects—(No of the popn)	GATT− Phaco	GATT	*p*-value
GATT-Phaco (58)	IOP (mmHG) = 26.40 ± 6.37 MED = 3.12 ± 0.80	IOP (mmHG) = 16.08 ± 2.38 MED =0.45 ± 0.96	IOP = 39.1% MED = −2.67	Microhyphema	21	28	0.5812
				Macrohyphema	25	25	0.5860
GATT (66)	IOP (mmHG) = 27.54 ± 8.09 MED = 3.35 ± 0.64	IOP (mmHG) = 15.50 ± 3.40 MED = 0.95 ± 1.50	IOP = 43.7% MED = −2.4	IOP spikes	10	36	0.00002
				Supracilliary effusion	15	26	0.1284
3.3.4. Comparison of Gonioscopy-assisted Transluminal Trabeculotomy Versus Trabeculectomy with Mitomycin C in Patients with Open-angle Glaucoma [26]							
Study type: Retrospective, single-center, comparative cohort study				Duration of study: 18 months			
No of Centers: 1				Total Population: 110 eyes—GATT: 61, TRAB: 49			
	Pre-op	Post-op	% Decrease	Adverse effects—(% of the popn)	GATT	TRAB	*p*-value
GATT (61)	IOP (mmHg) = 27.59 ± 4.70 MED = 2.92 ± 0.91	IOP (mmHG) = 15.26 ± 3.47 MED = −2.1 ± 1.5	IOP = 44.8% MED = −0.82	Hypotony	0	6	0.007
				Hyphaema	7	2	0.294
TRAB (49)	IOP (mmHg) = 30.04 ± 7.5 MED = 3.08 ± 0.73	IOP (mmHg) = 12.48 ± 4.58 MED = −2.3 ± 1.4	IOP = 58.5% MED = −0.78	IOP Spike	7	7	0.776

Abbreviations: GATT—Gonioscopy-assisted Transluminal Trabeculectomy; CS—cataract surgery; Phaco/P—phacoemulsification; IOP—intraocular pressure; MED—medication; Popn—population.

**Table 4:** Mechanism of action and recommended implantation technique of each device.

Device	Mechanism of Action	Implantation Technique
Hydrus Microstent	Expands and maintains Schlemm’s canal, increasing aqueous humor outflow.	Placed in Schlemm’s canal via a small corneal incision; covers 90° of the canal. Requires gonioscopy for angle visualization. Requires a separate corneal incision.
iStent	Bypasses trabecular meshwork to facilitate aqueous humor drainage into Schlemm’s canal.	Inserted through a corneal incision into Schlemm’s canal to bypass the trabecular meshwork. Minimal disruption to ocular structures. No new incisions are made.
GATT	Uses microcatheters to open the trabecular meshwork circumferentially—no physical device implantation.	Uses a microcatheter to open 360° of Schlemm’s canal via gonioscopy; more invasive with circumferential access. No physical device implanted, but trabecular meshwork is permanently incised.

## Data Availability

The dataset is available on request.
